# Retraction: Application of artificial intelligence-assisted Traditional Chinese medicine formula granules in bone and joint degenerative diseases in the field of holistic health

**DOI:** 10.3389/fmed.2026.1792278

**Published:** 2026-01-27

**Authors:** Frontiers Editorial Office

The Journal retracts the 2025 article cited above.

Following publication, the publisher has found evidence of peer review manipulation and non-existent or irrelevant citations. The investigation of these concerns resulted in the conclusion that the data, discussion, and methodology do not adequately support the conclusions of this article. As the scientific integrity of the article cannot be guaranteed and adhering to the recommendations of the Committee on Publication Ethics (COPE), the publisher therefore retracts the article.

This retraction was approved by the Chief Editors of Frontiers in Medicine and the Chief Executive Editor of Frontiers. The authors received a communication regarding the retraction and had a chance to respond. This communication has been recorded by the publisher.

